# Structural alterations of the intestinal epithelial barrier in Parkinson’s disease

**DOI:** 10.1186/s40478-015-0196-0

**Published:** 2015-03-10

**Authors:** Thomas Clairembault, Laurène Leclair-Visonneau, Emmanuel Coron, Arnaud Bourreille, Séverine Le Dily, Fabienne Vavasseur, Marie-Françoise Heymann, Michel Neunlist, Pascal Derkinderen

**Affiliations:** Inserm U913, 1 rue Gaston Veil, Nantes, F-44035 France; University Nantes, Nantes, F-44093 France; CHU Nantes, Institut des Maladies de l’Appareil Digestif, Nantes, F-44093 France; Inserm, CIC-04, Nantes, F-44093 France; CHU Nantes, Service d’Anatomie Pathologique, Nantes, F-44093 France; Inserm, UMR957, Nantes, F-44093 France; CHU Nantes, Department of Neurology, Nantes, F-44093 France

**Keywords:** Parkinson’s disease, Intestinal epithelial barrier, Enteric nervous system, Tight junctions, Occludin, ZO-1

## Abstract

Functional and morphological alterations of the intestinal epithelial barrier (IEB) have been consistently reported in digestive disorders such as irritable bowel syndrome and inflammatory bowel disease. There is mounting evidence that Parkinson’s disease (PD) is not only a brain disease but also a digestive disorder. Gastrointestinal involvement is a frequent and early event in the course of PD, and it may be critically involved in the early development of the disease. We therefore undertook the present survey to investigate whether changes in the IEB function and/or morphology occur in PD. Colonic biopsies were performed in 31 PD patients and 11 age-matched healthy controls. The para- and transcellular permeability were evaluated by measuring sulfonic acid and horseradish peroxidase flux respectively, in colonic biopsies mounted in Ussing chambers. The expression and localization of the two tight junctions proteins ZO-1 and occludin were analyzed by Western blot and immunofluorescence, respectively. The para- and transcellular permeability were not different between PD patients and controls. The expression of occludin, but not ZO-1, was significantly lower in colonic samples from PD patients as compared to controls and the cellular distribution of both proteins was altered in colonic mucosal specimens from PD patients. Our findings provide evidence that the IEB is morphologically altered in PD and further reinforce the potential role of the gastrointestinal tract in the initiation and/or the progression of the disease.

**Electronic supplementary material:**

The online version of this article (doi:10.1186/s40478-015-0196-0) contains supplementary material, which is available to authorized users.

## Introduction

The intestinal epithelium forms a regulated barrier, known as intestinal epithelial barrier (IEB), between the blood circulation and the contents of the intestinal lumen [1]. It prevents the passage of noxious contents while allowing the absorption and secretion of nutrients [1]. Penetration of this barrier occurs via two routes, either between epithelial cells via the paracellular pathway, or through epithelial cell via the transcellular pathway [1]. Among the most important structures of the intestinal barrier are the epithelial tight junctions (TJs) that connect adjacent enterocytes together to determine paracellular permeability through the lateral intercellular space [2]. They are formed by transmembrane proteins such as claudins and occludins connected to the actin cytoskeleton via high molecular weight proteins called zona occludens (ZO-1, ZO-2 and 3) [2]. Increased permeability of the IEB along with changes in the expression levels of TJs proteins have been consistently reported in several digestive disorders such as inflammatory bowel disease [3,4] and irritable bowel syndrome [5,6].

It has become evident over the last 20 years that PD is a gut disorder (reviewed in [7]). Gastrointestinal symptoms occur in almost every PD patient at some point and are among the most debilitating non-motor features of the disease [8]. These clinical data have been supported by *post mortem* studies that demonstrated the presence of Lewy bodies and neurites in the enteric neurons in nearly every case examined pathologically [9,10]. The German pathologist Heiko Braak suggested that the appearance of Lewy pathology in enteric neurons develop early in the course of disease, prior to the involvement of the central nervous system [11]. This led him to suggest that the gastrointestinal tract might be a portal of entry for a putative pathogen that would breach the IEB to induce the formation of Lewy bodies and neurites in the enteric neurons [11].

The high prevalence of gastrointestinal symptoms and pathology in PD and the possible derangement of gastrointestinal permeability in the pathogenesis of the disease prompted several groups to investigate IEB permeability in parkinsonian patients. The three studies, which have been carried out to date have all used absorption of sugar probes as a means to investigate non-invasively the paracellular permeability [12]. These studies, which have included a small number of patients, led to conflicting results. Two studies found a pattern of sugar absorption reminiscent of small intestine hyperpermeability in a subset of patients [13,14] while the third one showed an increase in sucralose excretion without changes in the lactulose/mannitol ratio, a pattern consistent with increased colonic permeability [15]. We therefore undertook the present research to analyze in more details the IEB in PD. To this end, a functional and structural characterization of the IEB was performed in colonic biopsies from PD patients.

## Materials and methods

### Subjects

A total of 42 subjects participated in this study, 31 PD patients and 11 healthy controls. PD patients aged 43–74 years were recruited from the movement disorder clinic at Nantes University Hospital, France. Diagnosis of PD was made according to criteria provided by the United Kingdom Parkinson’s Disease Survey Brain Bank [16]. Collected demographic data included gender, age at onset and disease duration, as well as age at colonoscopy. Complete drug history was obtained, and an approximation of the cumulative dose of L-dopa was made based on the equation developed by Parkkinen and collaborators [17]. Control subjects were healthy subjects who had a normal colonoscopy performed for colorectal cancer screening. All controls subjects underwent a detailed neurological examination to rule out PD symptoms and cognitive deficiency. Controls and PD patients were excluded if they suffered from irritable bowel syndrome and/or anorectal dysfunction. The study protocol was approved by the local Committee on Ethics and Human Research (Comité de Protection des Personnes Ouest VI) and registered on ClinicalTrials.gov (identifier NCT01748409). Written informed consent was obtained from each patient and from each normal volunteer according to the principles of Helsinki.

### Endoscopic procedure and colonic biopsies

For each subject, nine biopsies were taken in the sigmoid/descending colon during the course of a rectosigmoidoscopy for PD patients and during a colonoscopy for control subjects. Five biopsies were immersed in 4°C Hank’s Balanced Salt Solution (Life Technologies, Saint Aubin, France): three of these biopsies were immediately processed for the assessment of para- and transcellular permeability in Ussing chambers while the two other biopsies were used for immunohistochemistry experiments. Two biopsies were stored at −80°C in lysis buffer RA1 (Macherey-Nagel, Hoerdt, France) with 1% (v/v) β-mercaptoethanol (Sigma, Saint Quentin Fallavier, France) for further analysis by immunoblotting. The two remaining biopsies were snap frozen in liquid nitrogen at the time of collection and kept at −80°C.

### Para- and transcellular permeability of colonic biopsies in Ussing chambers

Three biopsies were mounted in Ussing chambers (World Precision Instruments; WPI, Hertfordshire, UK) exposing a surface of 0.011 cm^2^. Tissues were bathed on each side with 3 ml of F12 supplemented Dulbecco’s Modified Eagle medium (Invitrogen, France) containing 0.1% (v/v) fetal bovine serum, 200 mM Glutamine and 45 g/L of NaHCO_3_. The medium was continuously oxygenated and maintained at 37°C by a gas flow (95% O_2_/5% CO_2_). After a 30 min baseline period, 275 μL of apical medium was replaced with 200 μL of media containing 1 mg/mL of fluorescein-5,6-sulfonic acid (molecular weight: 400 Da) (Life Technologies) for a final concentration of 0.1 mg/mL to assess paracellular permeability. Seventy-five microliters of media with 10 mg/mL of Horse Radish Peroxydase (HRP) (Sigma) were also added to the basolateral chamber for a final concentration of 0.375 mg/mL to measure transcellular permeability in a subset of PD patients and control subjects. The fluorescence level of basolateral aliquots of 150 μl, reflecting paracellular transit from the luminal surface was measured every 30 min over a 3-hour period using a fluorimeter (Varioskan®, ThermoFisher Scientific, Cillebon sur Yvette, France). HRP quantities in the basolateral chamber, reflecting transcellular transit from the apical surface, was measured using an enzymatic activity assay with 3,3’,5,5’-tetramethylbenzidine reagent (BD Bioscience, Le Pont de Claix, France). Paracellular and transcellular permeabilities were determined by averaging the gradient of change in fluorescence intensity over time in the three biopsies that were analyzed per patient, using a linear regression fit model (GraphPad Prism 5, La Jolla, USA*).*

### Western blot

For the analysis of ZO-1 expression, total proteins from the 2 biopsies stored in RA1 buffer were precipitated and prepared for Polyacrylamide Gel Electrophoresis (PAGE) using protein precipitator and resuspension buffer (Protein solving buffer and (tris(2-carboxyethyl)phosphine) TCEP reducing agent, PSB/TCEP) from NucleoSpin Triprep Kit (Macherey-Nagel, Hoerdt, France) according to the manufacturer’s instructions. For experiments on the transmembrane protein occludin, the two dry frozen biopsies stored at −80°C were lysed in UTC buffer (7 M Urea, 2 M Thiourea and 4% CHAPS) containing a protease inhibitor cocktail (Complete™, Roche, Meylan, France) using the “Precellys 24” tissue homogenizer (Bertin technologies, Saint Quentin-en-Yvelines, France) and followed by sonication with “vibracell 75 186” device (Sonics, Newton CT, USA). Equal amounts of lysate were separated using the Invitrogen NuPage Novex 3-8% Tris-Acetate Midi Protein Gels™ for ZO-1 or NuPage Novex 4-12% Bis-Tris MidiGels™ for occludin before electrophoretic transfer to nitrocellulose membranes with the iBlot™ Dry Blotting System also from Invitrogen. Membranes were processed for immunoblotting using rabbit polyclonal anti-ZO-1 (1:500, Life Technologies) and rabbit anti-occludin (1:250, Abcam, Paris, France) antibodies and the relevant immunoreactive bands were quantified as previously described [18].

### Microdissection and immunohistochemistry

Microdissection was performed as previously described [19] in two out of the nine biopsies taken per patient. Each whole-mount preparation of submucosa obtained from a single biopsy was permeabilized for 3 hours in phosphate buffered saline (PBS)/NaN_3_ containing 1% (v/v) Triton X-100 and 10% (v/v) horse serum and then incubated with antibodies against phosphorylated alpha-synuclein (1:5000, WAKO, Osaka, Japan) and PGP9.5 (1:10,000; Ultraclone Limited, UK). Each whole-mount preparation of mucosa was treated for 24 h with Sca*l*e A2 solution composed of 4 M urea, 10% (w/v) glycerol and 0.1% (v/v) Triton X100 [20] then incubated with rabbit polyclonal antibodies to ZO-1 (1:100, Life Technologies) and occludin (1:100, Abcam). Suitable secondary antibodies conjugated to Alexa Fluor 488 and 594 were used (Invitrogen, Cergy-Pontoise, France). Following incubation with the secondary antibodies, the mucosa samples were treated for 10 minutes with a solution of 0.3% (w/v) of Sudan Black B powder (Sigma) dissolved in 70% (v/v) ethanol, then washed extensively with PBS. Whole specimen of submucosa and mucosa were viewed under an Axio Zoom.V16 stereomicroscope (Zeiss, Marly Le Roi, France). All samples were deidentified and studied in a blinded manner. For the analysis of ZO-1 and occludin immunofluorescence, the percentage of morphologically normal crypts per biopsy was calculated and the following classification was used: ‘normal’, more than 2/3 of morphologically normal crypts; ‘mild disruption’: between 1/3 and 2/3 of morphologically normal crypts; ‘disrupted’: less than 1/3 of morphologically normal crypts.

### Statistics

All data are given as the mean ± standard error of the mean (SEM). For comparisons of means between groups, a Mann–Whitney test was performed. Differences were deemed statistically significant if p < 0.05.

## Results

A total of 31 PD patients and 11 controls were included. Table 1 shows the main clinical and demographic features of all patients. Age and sex did not differ significantly between PD patients and control subjects (mean age was 64.2 ± 2.1 for PD and 60.6 ± 1.4 for controls, p = 0.25; 22/31 male in PD group and 6/11 male in control group, p = 0.13).

**Table 1:** **Main clinical characteristics of PD patients**

	**Age**	**Sex**	**Disease duration (years)**	**Cumulative lifetime dose of L-dopa (mg)**
**1**	**70**	**F**	**2**	**0**
**2**	**64**	**M**	**NK**	**NK**
**3**	**70**	**M**	**NK**	**NK**
**4**	**66**	**M**	**23**	**66111000**
**5**	**47**	**F**	**2**	**0**
**6**	**62**	**M**	**4**	**3823375**
**7**	**74**	**M**	**13**	**1505625**
**8**	**53**	**M**	**4**	**2372500**
**9**	**69**	**M**	**2.5**	**273750**
**10**	**43**	**F**	**11**	**508050**
**11**	**67**	**M**	**2.3**	**0**
**12**	**58**	**M**	**7**	**2135250**
**13**	**58**	**M**	**9**	**1533000**
**14**	**72**	**F**	**14**	**1442662.5**
**15**	**64**	**M**	**4**	**492750**
**16**	**64**	**M**	**10**	**355875**
**17**	**56**	**M**	**NK**	**NK**
**18**	**66**	**M**	**4.6**	**0**
**19**	**62**	**M**	**26**	**1560375**
**20**	**70**	**F**	**2.2**	**219000**
**21**	**57**	**M**	**1.8**	**0**
**22**	**58**	**M**	**13**	**2007500**
**23**	**50**	**M**	**8**	**1286625**
**24**	**69**	**F**	**28**	**3759500**
**25**	**62**	**M**	**8**	**511000**
**26**	**56**	**F**	**8**	**1241000**
**27**	**46**	**M**	**10**	**1213625**
**28**	**56**	**F**	**8.4**	**866875**
**29**	**58**	**F**	**4.8**	**447125**
**30**	**54**	**M**	**24**	**3085423**
**31**	**58**	**M**	**10**	**3374130**

NK: not known.

### Para- and transcellular permeability are unaffected in PD

**Figure 1:**
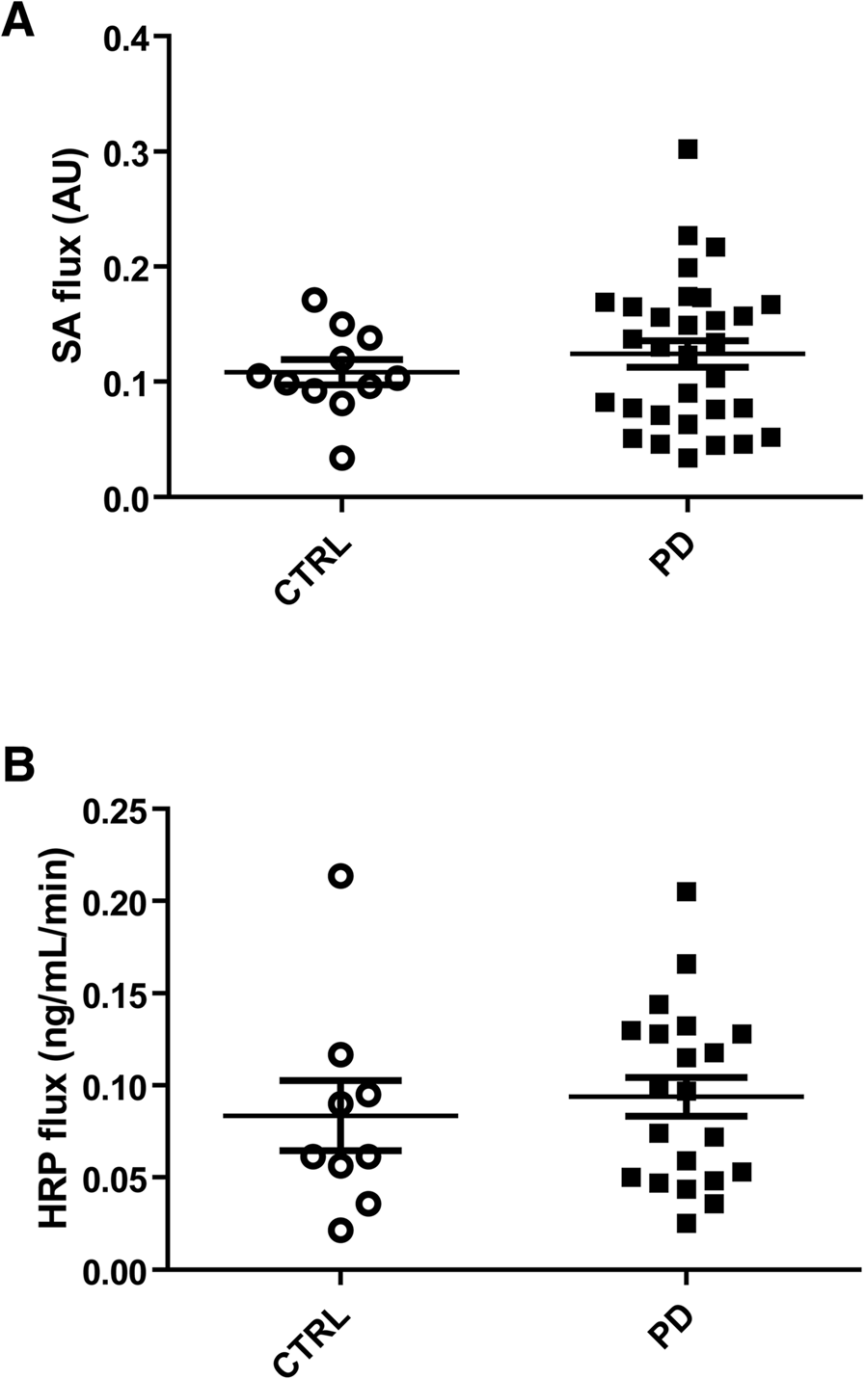
**Comparison of para- and transcellular permeability in PD patients and healthy controls. (A)** For the evaluation of paracellular permeability, the flux of sulfonic acid (SA flux) was measured in colonic biopsies mounted in Ussing chambers, expressed in arbitrary units (AU), in PD patients (n = 31) and controls (CTRL, n = 11). No significant changes were observed between the two groups (p = 0.65). **(B)** For the evaluation of paracellular permeability, the flux of horseradish peroxidase (HRP flux) was measured in colonic biopsies mounted in Ussing chambers, expressed in ng/mL/min, in PD patients (n = 21) and controls (CTRL, n = 9). No significant changes were observed between the two groups (p = 0.39).

In a first set of experiments, we evaluated whether IEB is functionally altered in PD patients. The para- and transcellular permeability of colonic biopsies were measured in Ussing chambers in both PD patients and control subjects using sulfonic acid and HRP, respectively. No difference in the sulfonic acid flux was observed between PD patients and control subjects (n = 31 and 11, respectively; p = 0.65) (Figure 1A). HRP flux was also comparable between PD subjects and healthy controls (n = 21 and 9, respectively; p = 0.39) (Figure 1B). Although not statistically different from controls, the sulfonic acid and HRP flux values were heterogeneous between PD patients (Figure 1A and B). We thus investigated if the main clinical features of the disease had any influence on IEB permeability. We did not observe any correlation between age, disease duration or lifetime cumulative dose of L-DOPA and the values of sulfonic acid or HRP flux (Table 2).

**Table 2:** **Spearman’s correlation with sulfonic acid (SA) and HRP permeability in PD patients**

	**Age**	**Disease duration**	**Cumulative lifetime dose of L-dopa**
**SA**	**p = 0.090**	**p = 0.788**	**p = 0.830**
	**r = 0.303**	**r = 0.052**	**r = −0.046**
**HRP**	**p = 0.165**	**p = 0.469**	**p = 0.754**
	**r = 0.323**	**r = 0.172**	**r = 0.082**

**Figure 2:**
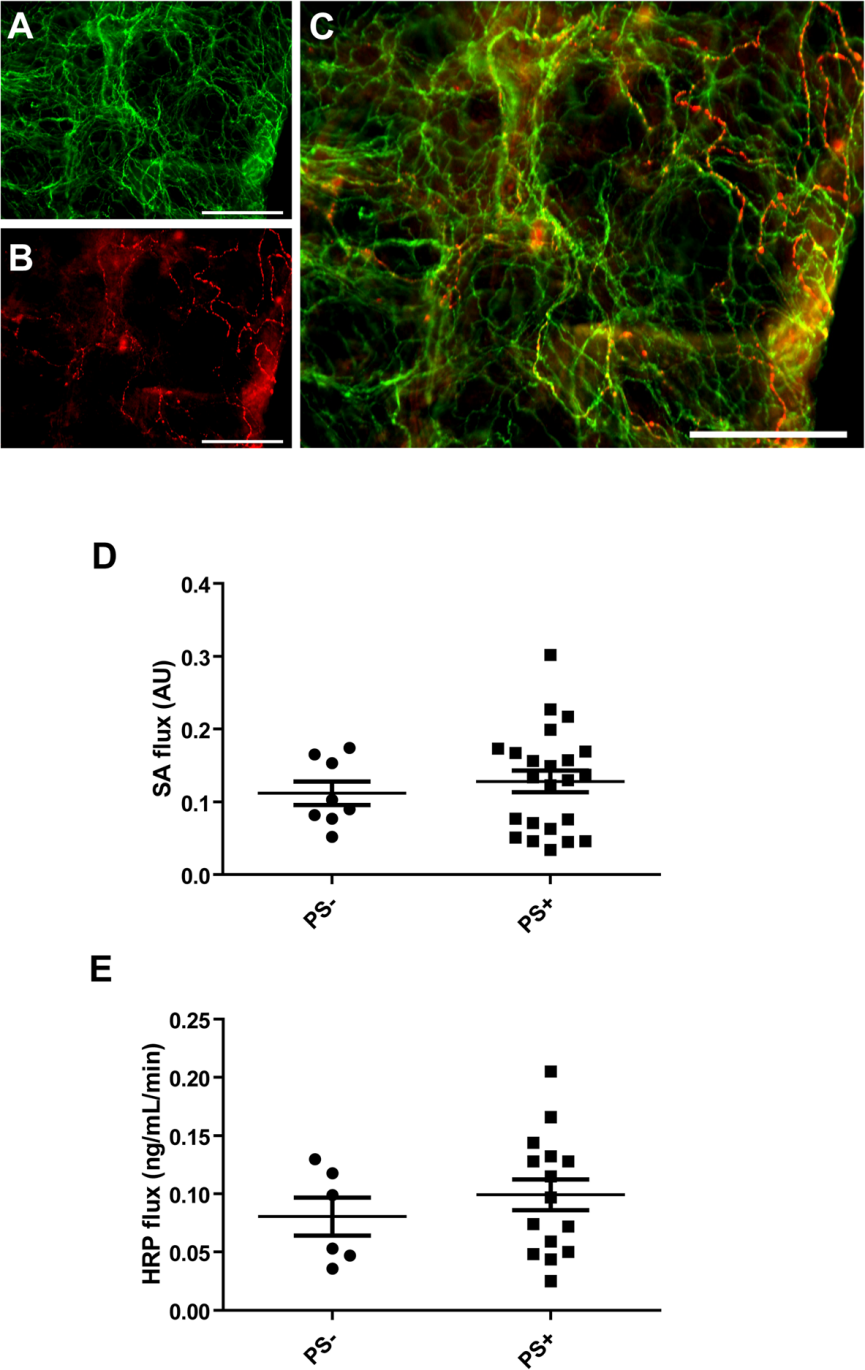
**Sulfonic acid and HRP flux in PD in patients with and without enteric Lewy pathology.** Representative photomicrograph depicting multiple Lewy neurites in a whole-mount of submucosa immunoreactive for PGP9.5 **(A)** that was also positive for phosphorylated alpha-synuclein **(B and C)** Scale bar: 200 μm. **(D)** The values of sulfonic acid flux was not different between PD patients with (PS+) or without (PS-) phospho-synuclein immunoreactive neurites (p = 0.93). **(E)** The values of HRP flux was not different between PD patients with (PS+) or without (PS-) phospho-synuclein immunoreactive neurites (p = 0.46).

There is a growing body of evidence supporting a key role for submucosal enteric neurons in the regulation of IEB functions [21-23]. This prompted us to study if the flux values of sulfonic acid and HRP were related to the presence of Lewy bodies and Lewy neurites in the submucosa. To this end, two biopsies per patient were immunohistochemically assessed for the presence of Lewy pathology using antibodies against phosphorylated alpha-synuclein and PGP9.5 (Figure 2A and B). A biopsy was deemed positive when containing at least one inclusion immunoreactive for both phosphorylated alpha-synuclein and PGP9.5 (Figure 2C). A patient was noted as positive when at least one of the two biopsies displayed inclusion(s). In accordance with our previous reports [24,25], the intraneuronal inclusions found in the submucosal plexus were chiefly observed in the neuronal processes and thus reminiscent of Lewy neurites (Figure 2A-C). Twenty-three out of 31 PD patients were positive for phosphorylated alpha-synuclein inclusions. All control subjects were devoid of inclusions. The values of sulfonic acid and HRP flux were not different between PD patients with or without inclusions (Figure 2D and E).

### Expression of the tight junction protein occludin is decreased in PD

**Figure 3:**
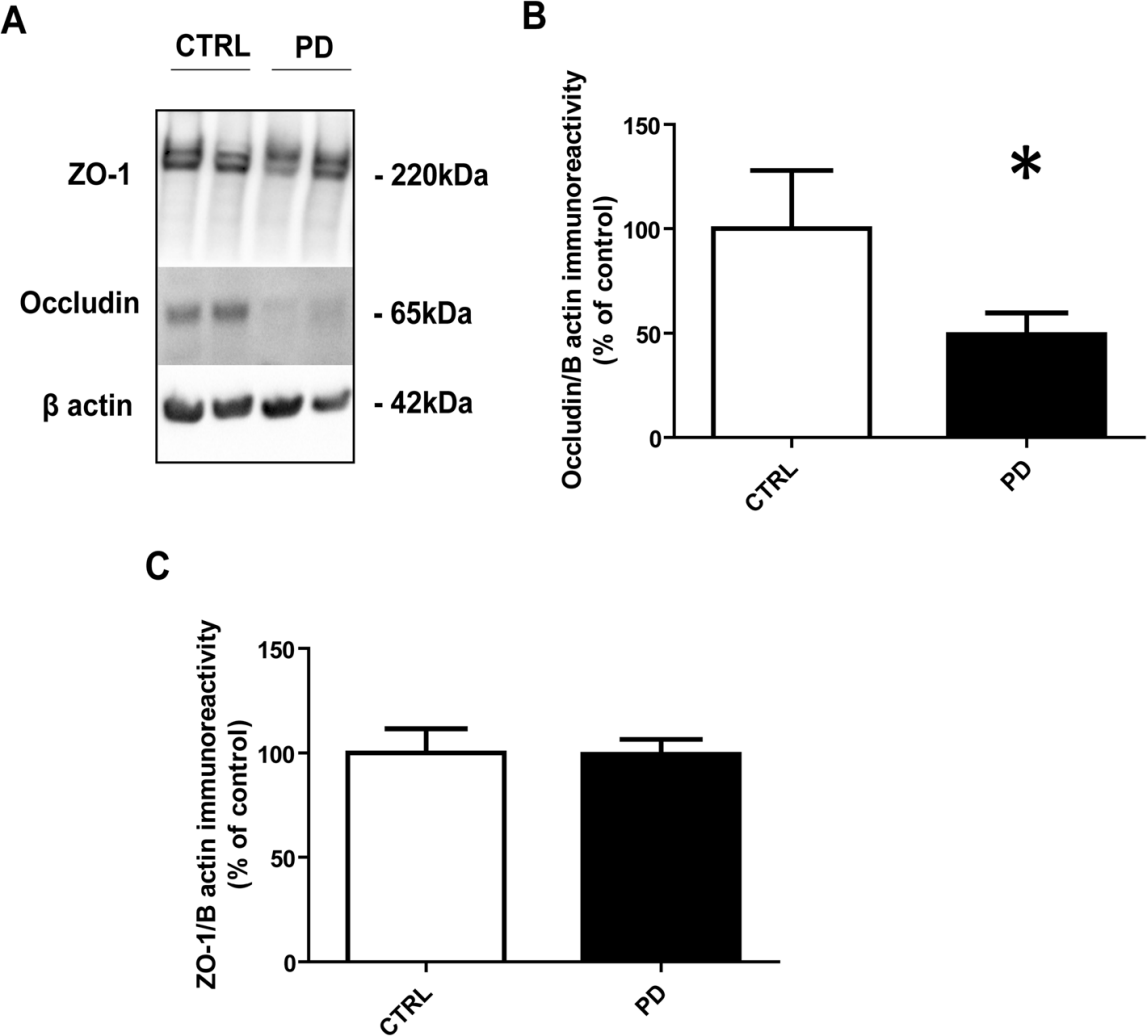
**Expression of TJs proteins in colonic biopsies from patients with Parkinson’s disease (PD) and control subjects (CTRL).** Biopsies lysates (20 μg of protein per sample) were subjected to immunoblot analysis using antibodies against occludin and ZO-1 **(A)**. Beta-actin was used as a loading control. The optical densities of occludin **(B)** and ZO-1 **(C)** immunoreactive bands were measured, normalized to the optical densities of beta-actin immunoreactive bands in the same samples and expressed as percentages of controls. Data correspond to mean ± SEM of 11 samples for control subjects (CTRL) and 31 samples for Parkinson’s disease (PD) patients. Patients versus control, *: p < 0.05.

We next investigated whether structural changes in the IEB occur in PD. The expression levels of the TJs proteins ZO-1 and occludin were analyzed by Western blot in colonic biopsies from PD subjects and healthy controls. A significant decrease in the expression of occludin was observed in colonic samples of PD patients as compared to controls (Figure 3A and B). A doublet band of approximately 220 kDa was observed on Western blot with antibodies against ZO-1 (Figure 3A). As previously shown, these two bands most likely represent the two ZO-1 isoforms [26,27]. By contrast to occludin, no change in ZO-1 expression levels was observed in PD whether the two bands were quantified together (Figure 3C) or separately (data not shown).

### Cellular distribution of the TJs proteins is altered in PD

**Figure 4:**
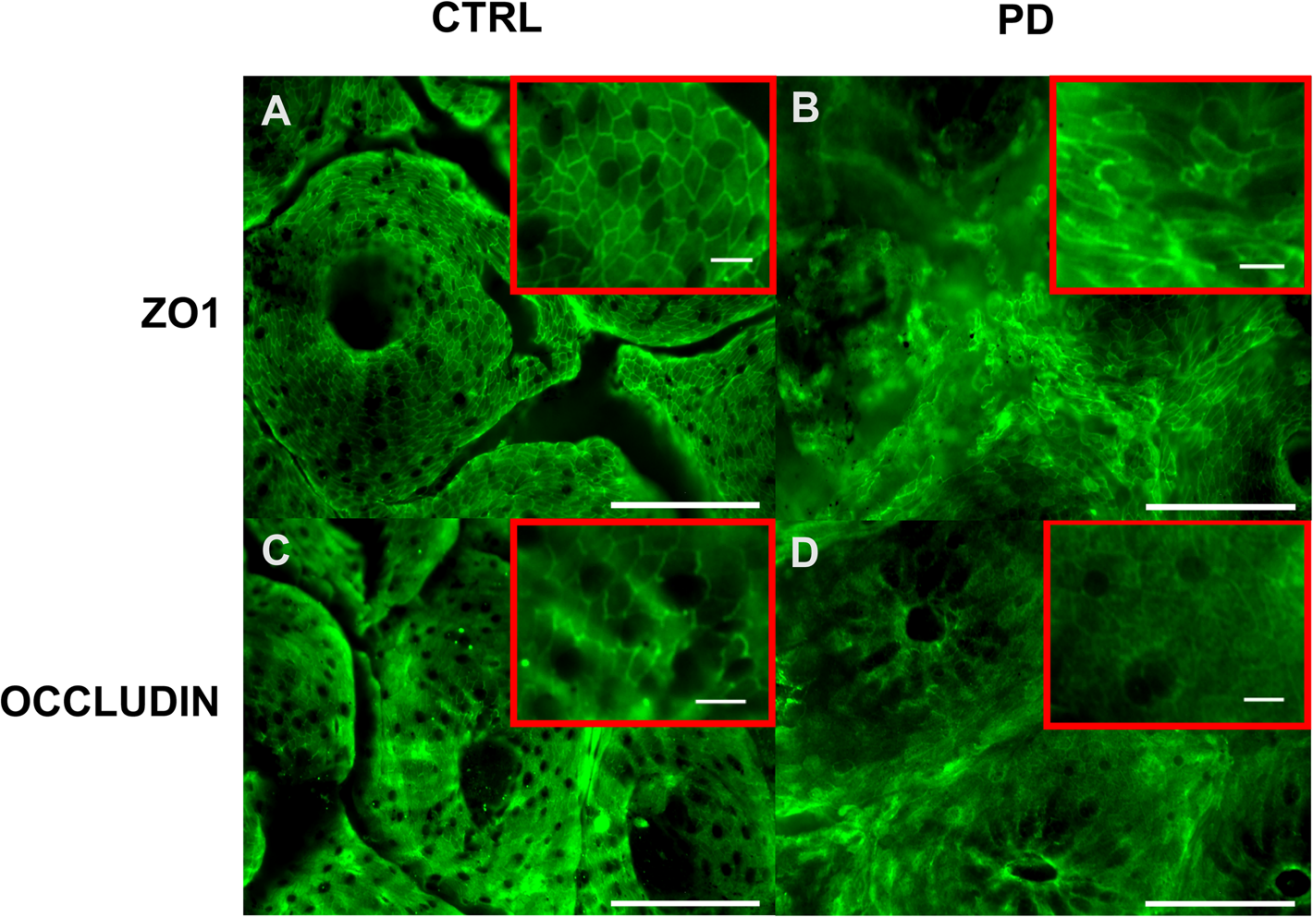
**Localization of TJs proteins in the colonic mucosa of healthy controls (CTRL) and patients with Parkinson’s disease (PD).** Representative photomicrographs of the colonic mucosa labeled with antibodies against ZO-1 **(A, B)** and occludin **(C, D)** in the colonic mucosa of control and PD patients; scale bar: 100 μm. High-magnification image of each area marked by red square; scale bar: 10 μm.

The cellular distribution of occludin and ZO-1 was further investigated by immunofluorescence in 8 controls and 31 PD subjects. Samples from 3 controls were excluded because the mucosa was too small and/or too damaged to allow a reliable analysis of the TJs morphology. A mean of 96.4 crypts per biopsy were analyzed. We observed differences in the cellular distribution of both ZO-1 and occludin between PD patients and controls (Figure 4). A normal and typical reticular pattern of occludin and ZO-1 staining was observed in the colonic samples of 6 out of 8 controls (Figure 4A and C, Additional file 1) and in only 9/31 PD patients (Figure 4B and D, Additional file 1). TJs morphology was disrupted and irregularly distributed in the mucosa of 1 out of 8 controls (Figure 4A and C, Additional file 1) and in 14/31 PD patients (Figure 4B and D, Additional file 1). An occasional and mild disruption of TJs morphology was observed in the remaining control subject and in 8/31 PD samples (Figure 4 and Additional file 1). An increased staining of occludin in the cytoplasm of colonic enterocytes, suggestive of protein internalization, was observed in PD samples as opposed to the healthy group where occludin was mostly located in the TJs (Figure 4C and D). Worthy of note was the presence of moderate to severe TJs disorganization in the 5 patients who had never received levodopa, suggesting that the altered TJs morphology was not related to chronic levodopa intake (Additional file 2 and Table 1).

## Discussion

The TJs are intercellular protein complex located at the apical portions of the lateral membranes of epithelial cells which play a key role in the regulation of IEB paracellular permeability. They are composed of transmembrane proteins, such as claudins and occludin and a wide spectrum of cytosolic proteins among which is ZO-1 [1]. By showing a decrease in occludin expression along with TJs disorganization, our study is the first to provide evidence that the IEB is structurally altered in PD. Previous studies have shown intestinal tissue expression and distribution of occludin to be markedly decreased in patients with intestinal permeability disorders, including inflammatory bowel disease [28] and irritable bowel syndrome [6]. Recent data from genetic and epidemiological studies provided support for an association between diseases of the gastrointestinal tract and the susceptibility to developing PD. The CARD15 gene known to be associated with Crohn's disease is over-represented in patients with PD [29]; *vice versa*, the Leucine-rich repeat kinase 2 (LRRK2) gene, a causative PD mutation, was recently identified as a major susceptibility gene for Crohn’s disease by genome-wide association studies [30]. Moreover, patients with irritable bowel syndrome are almost 50% more likely to develop PD than people who are free of this gastrointestinal disorder [31]. Our results show that the two disorders also share similarities at the molecular levels further supporting the assumption that irritable bowel syndrome may actually belong to early signs of gastrointestinal involvement in PD [32]. They also support the hypothesis that the brain gut-axis might be critically involved in the pathophysiology of both disorders [7,33].

Studies of intestinal permeability in humans have mainly been carried out with *in vivo* techniques, usually with oral ingestion of various sugar probes and measurement of urinary excretion [12]. To date, the three studies that attempted to evaluate intestinal permeability in PD using this technique have provided only preliminary and conflicting results. Two of these studies focused on the lactulose/mannitol ratio, which evaluate small intestinal permeability. As a group, the 15 PD patients studied by Davies and collaborators had a significant increase in the lactulose/mannitol ratio when compared to age and sex matched controls, but individual results in both groups were highly overlapping [14]. Salat-Foix *et al.* showed that the lactulose/mannitol ratio was only marginally higher in 3 out of 12 PD patients [13]. In addition to lactulose/mannitol ratio, Forsyth *et al.* also used sucralose absorption for the assessment of colon permeability in 9 PD patients and 10 controls. They did not observe any difference in the lactulose/mannitol ratio between the two groups but found a significantly greater permeability to sucralose in PD subjects [15]. The inconsistent results on intestinal permeability in PD obtained with sugar probes prompted us to measure IEB permeability by another technique, namely Ussing chambers, in a larger sample size. Although less commonly used than sugar absorption, the Ussing chambers has proven to be a reliable and effective tool to measure IEB permeability of gastrointestinal biopsies either paracellularly or transcellularly over a 3 hour period [34]. Using this approach, we showed that there were no significant differences in para- and transcellular permeabilities between PD subjects and controls. Nevertheless, the values of paracellular permeability as assessed by the sulfonic acid flux in Ussing chamber were highly heterogeneous between PD patients, some displaying a level comparable to controls while others had a more than a 2.5 fold increase in sulfonic acid flux. These data suggest that increased colonic permeability may be a feature for a subset of PD patients, as already reported when sugar probes were used [15]. The factors responsible for this heterogeneity still remain to be determined, as we did not observe any correlation between age, cumulative dose of L-Dopa disease duration and the severity of altered permeability.

On the surface, our results may seem contradictory, as the decreased expression of occludin observed in PD patients was not accompanied by changes in paracellular permeability. Occludin is a tetraspan protein with two extracellular loops, which homophilically interact with the adjacent cells [2]. Its role on IEB has been debated since its initial discovery in 1993 [35]. Initial studies strongly suggested that occludin was not required for the TJs formation or the maintenance of barrier function as occludin knockout mice lacked any noticeable defect in intestinal TJs morphology or barrier function [36]. This has been recently challenged in an elegant study published by Al-Sadi *et al.* [37]. The purpose of their research was to better delineate the involvement of occludin in IEB by studying the transepithelial flux of various-sized probes after knocking down occludin both *in vitro* and *in vivo*. They showed that the occludin knock down caused a marked increase in the flux rates of macromolecules above 5 kDa such as inulin and dextran but had only modest effect on flux of smaller-sized probes under 200 Da such as mannitol and urea [36]. Fluorescein-5,6-sulfonic acid, which was used for the assessment of paracellular permeability in our study has a molecular weight of 400 Da, likely to be too small for detecting defects in IEB permeability induced by a mere down regulation of occludin. This may explain the lack of significant changes in IEB permeability observed in PD patients in our study in spite of the occurrence of structural changes.

The question arises as to what might be the clinical relevance of our experimental findings. A current theory, the so-called Braak’s theory, assumes that PD originates in the gastrointestinal tract [11]. Braak and co-workers suggested that the appearance of Lewy pathology occurs in the earliest stage of PD in both the enteric nervous system and the dorsal motor nucleus of the vagus [11,38]. This led Braak to postulate that a pathogen may breach the IEB to trigger Lewy pathology in the terminal axons of the enteric neurons, further spreading to the central nervous system via the vagal preganglionic innervation of the gut [11,39]. In light of these considerations, our results demonstrating altered intestinal TJs structure in PD gain in importance as the down regulation of occludin may favor the entry of a putative pathogen. This must be however balanced, as the stomach in contrast to the colon appears to be the most suitable target for the pathologic insult to occur in Braak’s scenario. Several studies have indeed described that Lewy pathology is distributed following a rostro-caudal gradient in PD, with the lower esophagus and stomach having the greatest involvement and the colon and rectum the lowest [9,10], a distribution that parallels the vagal innervation of the gastrointestinal tract [40]. Further studies are therefore warranted to analyze the mucosal barrier permeability and morphology in gastric and duodenal samples from PD patients.

## Conclusions

In conclusion, we provide evidence for the first time that morphological changes in the IEB occur in PD patients. Our results further reinforce the possible role of the gastrointestinal tract in the pathophysiology of PD. Further work is needed to determine if occludin down regulation in the gut might facilitate the spreading of PD pathology in the enteric nervous system and in the brain.

## Additional files

Additional file 1:
**Percentage of morphologically normal crypts in the colonic mucosa of healthy controls (CTRL) and patients with Parkinson’s disease (PD).**
Additional file 2:
**Localization of TJs proteins in the colonic mucosa of the 5 with Parkinson’s disease (PD) who had never received levodopa.** Scale bar: 100 μm.
