# Supplementary figures and images for: Structural alterations of the intestinal epithelial barrier in Parkinson’s disease
Source: Acta Neuropathol Commun. 2015 Mar 10;3:12. doi: 10.1186/s40478-015-0196-0 (PMC4353469; doi:10.1186/s40478-015-0196-0)

# Supplementary figure 1

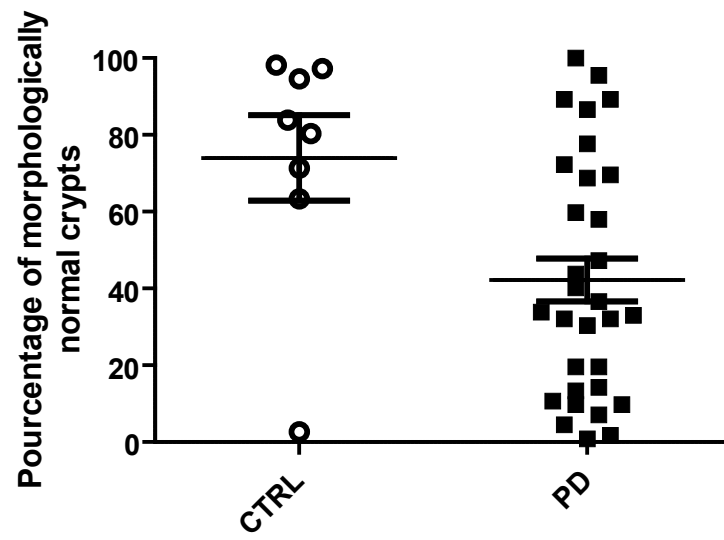

Supplement: Additional file 1: — Percentage of morphologically normal crypts in the colonic mucosa of healthy controls (CTRL) and patients with Parkinson’s disease (PD). [file 40478_2015_196_MOESM1_ESM.pdf]

Supplementary figure 2

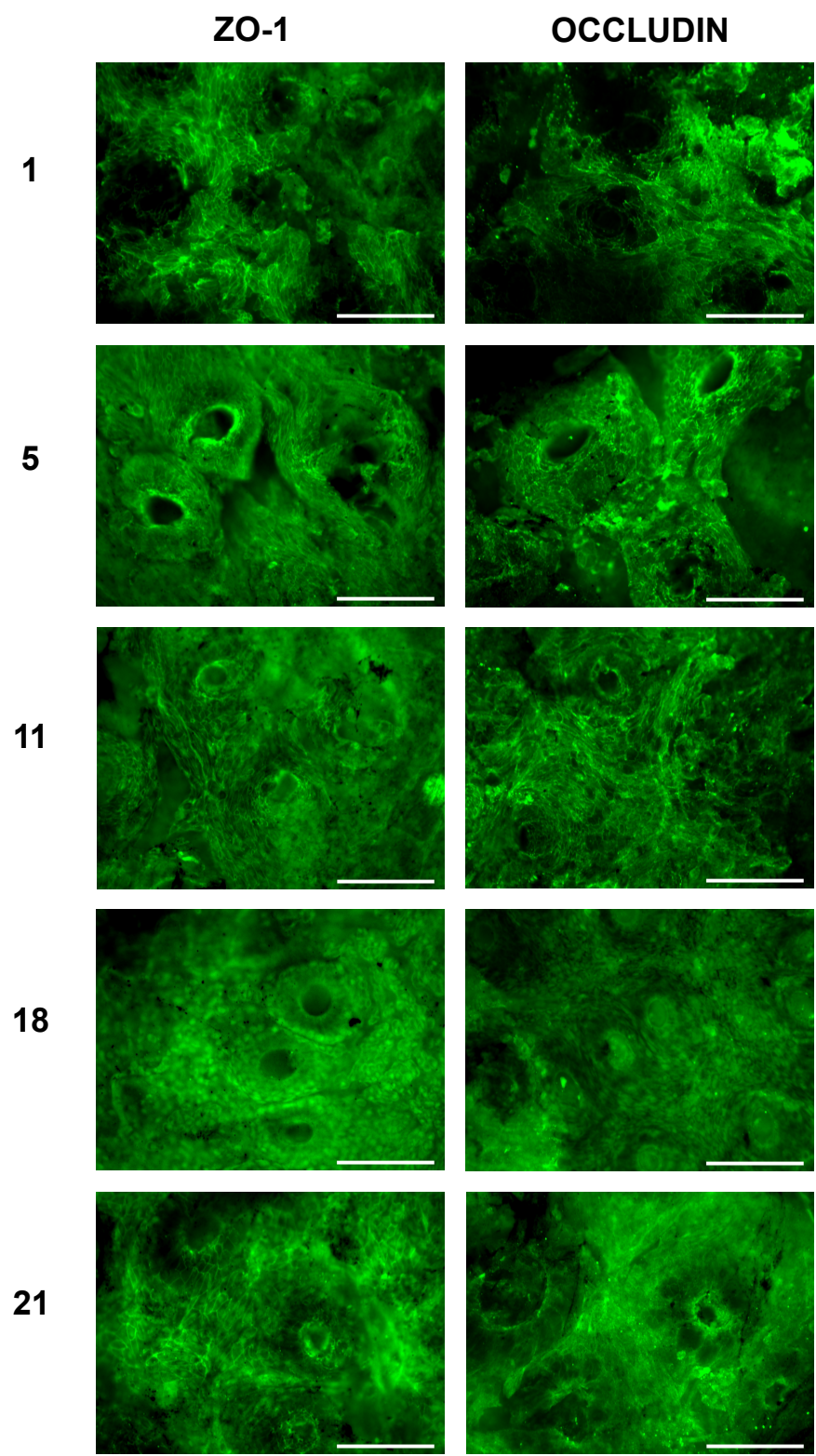

Supplement: Additional file 2: — Localization of TJs proteins in the colonic mucosa of the 5 with Parkinson’s disease (PD) who had never received levodopa. Scale bar: 100 μm. [file 40478_2015_196_MOESM2_ESM.pdf]
